# Changes in H^+^-ATP Synthase Activity, Proton Electrochemical Gradient, and pH in Pea Chloroplast Can Be Connected with Variation Potential

**DOI:** 10.3389/fpls.2016.01092

**Published:** 2016-07-22

**Authors:** Vladimir Sukhov, Lyubov Surova, Ekaterina Morozova, Oksana Sherstneva, Vladimir Vodeneev

**Affiliations:** Department of Biophysics, N. I. Lobachevsky State University of Nizhny NovgorodNizhny Novgorod, Russia

**Keywords:** electrochromic pigment absorbance shifts, H^+^-ATP synthase, light scattering, photosynthesis, proton motive force, variation potential

## Abstract

Local stimulation induces generation and propagation of electrical signals, including the variation potential (VP) and action potential, in plants. Burning-induced VP changes the physiological state of plants; specifically, it inactivates photosynthesis. However, the mechanisms that decrease photosynthesis are poorly understood. We investigated these mechanisms by measuring VP-connected systemic changes in CO_2_ assimilation, parameters of light reactions of photosynthesis, electrochromic pigment absorbance shifts, and light scattering. We reveal that inactivation of photosynthesis in the pea, including inactivation of dark and light reactions, was connected with the VP. Inactivation of dark reactions decreased the rate constant of the fast relaxation of the electrochromic pigment absorbance shift, which reflected a decrease in the H^+^-ATP synthase activity. This decrease likely contributed to the acidification of the chloroplast lumen, which developed after VP induction. However, VP-connected decrease of the proton motive force across the thylakoid membrane, possibly, reflected a decreased pH in the stroma. This decrease may be another mechanism of chloroplast lumen acidification. Overall, stroma acidification can decrease electron flow through photosystem I, and lumen acidification induces growth of fluorescence non-photochemical quenching and decreases electron flow through photosystem II, i.e., pH decreases in the stroma and lumen, possibly, contribute to the VP-induced inactivation of light reactions of photosynthesis.

## Introduction

Local stimulation rapidly elicits systemic responses in plants (Gallé et al., 2015), including changes in gene expression (Stanković and Davies, 1996; Fisahn et al., 2004) and phytohormone production (Dziubinska et al., 2003; Hlaváčková et al., 2006; Hlavinka et al., 2012), increases in plant resistance to stressors (Retivin et al., 1997, 1999; Sukhov et al., 2014b, 2015a; Surova et al., 2016), the activation of respiration (Dziubinska et al., 1989; Filek and Kościelniak, 1997), etc. Numerous works have described the influence of local stimuli on photosynthetic processes (Hlaváčková et al., 2006; Krupenina and Bulychev, 2007; Grams et al., 2009; Pavlovič et al., 2011; Hlavinka et al., 2012; Sukhov et al., 2012, 2014a,b, 2015a,b; Vredenberg and Pavlovič, 2013; Bulychev and Komarova, 2014; Sherstneva et al., 2015, 2016; Surova et al., 2016), including reduced CO_2_ assimilation, decreases in the photosystem I (PSI) and photosystem II (PSII) quantum yields, the growth of fluorescence non-photochemical quenching (NPQ), and the activation of cyclic electron flow. Electrical signals, namely the action potential (AP), which is mainly induced by non-damaging stimuli, and the variation potential (VP), which is mainly caused by damaging stimuli, are the most likely links between stimulated and non-stimulated zones during the systemic responses of plants (Sukhov, 2016).

The AP is a self-propagating electrical signal that is primarily related to passive ions fluxes, including Ca^2+^, Cl^-^, and K^+^ fluxes (Beilby, 1984, 2007; Dziubinska, 2003; Krol et al., 2004; Felle and Zimmermann, 2007; Sukhov et al., 2011). The VP is a local electrical reaction to hydraulic and/or chemical signal propagation (Malone, 1994; Stahlberg and Cosgrove, 1996; Mancuso, 1999; Vodeneev et al., 2012, 2015; Sukhov et al., 2013). Transient H^+^-ATPase inactivation is the main mechanism of VP generation (Julien et al., 1991; Stahlberg and Cosgrove, 1997; Vodeneev et al., 2015), but ion fluxes also participate in the reaction (Julien et al., 1991; Vodeneev et al., 2011, 2015; Katicheva et al., 2014). According to studies of Chara alga by Krupenina and Bulychev (2007), Bulychev and Komarova (2014), the influence of AP on photosynthesis is likely a function of Ca^2+^ flux into cell. However, the influence of VP on photosynthesis in higher plants likely involves another mechanism. Numerous works (Grams et al., 2009; Sukhov et al., 2014a; Sherstneva et al., 2015, 2016) have reported that the VP-connected H^+^ influx is a potential mechanism of photosynthetic inactivation.

Variation potentials appear to affect photosynthesis in different ways (Sukhov, 2016). Inactivation of dark reactions of photosynthesis is an important mechanism of the photosynthetic response (Sukhov et al., 2012, 2014a,b, 2015b; Sherstneva et al., 2015), and a decreased flow of CO_2_ into mesophyll cells is likely responsible for photosynthesis inactivation (Gallé et al., 2015; Sukhov, 2016). This decreased flux can result from an increase in the ${HCO}_{3}^{-}$:CO_2_ ratio in the apoplast, the inactivation of aquaporins, or changes in carbonic anhydrase activity (Grams et al., 2009; Gallé et al., 2013; Sukhov et al., 2014a; Sherstneva et al., 2015). These decreases in the CO_2_ flow can all be associated with the changes in cytoplasmic acidification and apoplastic alkalization observed during VP generation (Grams et al., 2009; Sukhov et al., 2014a; Sherstneva et al., 2015, 2016).

Changes in parameters of light reactions of photosynthesis can be also observed after propagation of electrical signals (Krupenina and Bulychev, 2007; Grams et al., 2009; Pavlovič et al., 2011; Sukhov et al., 2012, 2014a,b, 2015a,b; Vredenberg and Pavlovič, 2013; Sherstneva et al., 2015, 2016; Surova et al., 2016). These changes demonstrate that electrical signals influence the thylakoid membrane (Sukhov, 2016), and the influence may be connected with inactivation of dark reactions of photosynthesis, an increase in the ATP:ADP ratio in the chloroplast stroma, and the inactivation of H^+^-ATP synthase (Pavlovič et al., 2011; Sukhov et al., 2012, 2014a; Sukhov, 2016). Furthermore, the VP can decrease electron flow through the acceptor side of PSI (Sukhov et al., 2012) and increase NPQ (Sukhov et al., 2014a) independently of inactivation of dark reactions of photosynthesis. The last responses may be related to pH changes in the chloroplast stroma and lumen (Sukhov et al., 2014b, 2015a; Sukhov, 2016), but this relationship has not yet been experimentally investigated. Thus, an experimental investigation of the influence of the VP on the H^+^-ATP synthase activity, proton gradient across the thylakoid membrane, and pH in the chloroplast is important to understand the mechanism underlying the photosynthetic response.

The registration of changes in green light absorption by photosynthetic pigments, including the ‘electrochromic pigment absorbance shift’ (ECS) and ‘light scattering’ (LS), are classical, widely used, non-invasive methods used to investigate electrical and proton gradients across thylakoid membranes (Deamer et al., 1967; Murakami and Packer, 1970; Ivanov et al., 2001; Avenson et al., 2004; Schreiber and Klughammer, 2008; Bailleul et al., 2010; Klughammer et al., 2013; Wang et al., 2015). It should be noted that ECS and LS are often measured in intact leaves or in segments of leaves (Kramer and Crofts, 1989; Sacksteder et al., 2000; Ruban et al., 2002; Schreiber and Klughammer, 2008; Klughammer et al., 2013; Wang et al., 2015).

The ECS is a change in the leaf absorbance between 515 and 525 nm, considered to be proportional to the electrical potential across the thylakoid membrane, and associated with carotenoids and Chl b (Schreiber and Klughammer, 2008; Klughammer et al., 2013). The proton electrochemical gradient (proton motive force, pmf), transmembrane electrical potential (ΔΨ), and proton gradient (ΔpH) can be estimated using the ECS relaxation after the ‘light-dark’ transition (Avenson et al., 2004; Schreiber and Klughammer, 2008; Bailleul et al., 2010; Klughammer et al., 2013). This relaxation can also be used to calculate the H^+^-ATP synthase activity (Morita et al., 1982; Kramer and Crofts, 1989; Sacksteder et al., 2000; Klughammer et al., 2013; Wang et al., 2015).

Light scattering is a change in the leaf absorbance at approximately 535 nm that is characterized by slow relaxation kinetics (minutes), independently from the ECS (Schreiber and Klughammer, 2008). LS is caused by the internal acidification of thylakoids upon light-induced ΔpH formation (Deamer et al., 1967; Schreiber and Klughammer, 2008); this relationship is supported by the monotonous growth of LS in response to pH decreases in the physiological range (Deamer et al., 1967; Murakami and Packer, 1970). Aggregation of light harvesting complexes in thylakoids, which is connected with protonation of these complexes, is a probable mechanism for the shift in LS (Horton et al., 1991, 2005; Ruban et al., 2002). Therefore, LS reflects the luminal pH in the chloroplast and can be used as a semi-quantitative indicator of membrane energization (Schreiber and Klughammer, 2008).

The aims of this study were to investigate the influence of the VP on the electrical and proton gradients across thylakoid membranes, the H^+^-ATP synthase activity, and the pH of pea leaves (*Pisum sativum* L.).

## Materials and Methods

### Plant Material

Pea seedlings (14–21 days old) were used in this investigation. Seedlings were cultivated hydroponically in a Binder KBW 240 plant growth chamber (Binder GmbH, Tuttlingen, Germany) at 24°C, with a 16/8-h (light/dark) photoperiod. White light was used (∼100 μmol m^-2^ s^-1^).

### Burning and Measurements of Electrical Activity

**FIGURE 1:**
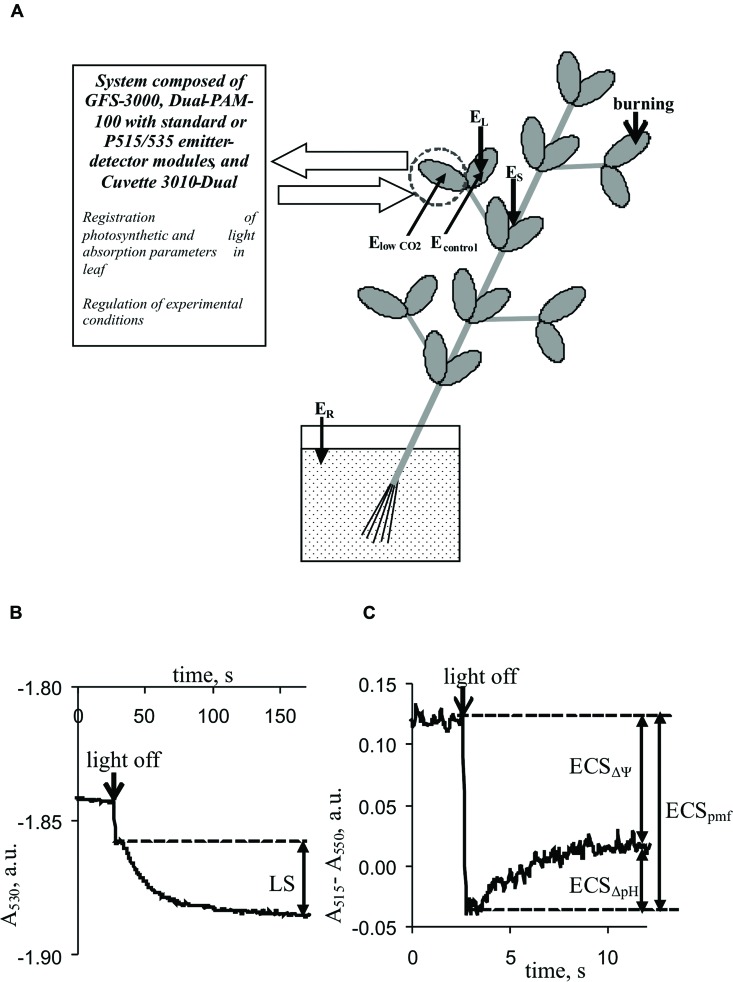
**Positions of burning (flame, 3–4 s, ∼1 cm^2^), electrical potential monitoring, and photosynthetic and light absorption parameter measurements in plants. (A)** *E_L_* and *E_S_*, Ag^+^/AgCl electrodes connected to the leaf and stem, *E_R_*, reference electrode; distance between *E_L_* and *E_S_*, 3–4 cm. E_lowCO_2__ and E_control_, electrodes from silver wire. **(B)** Kinetics of leaf light absorption at 530 nm, A_530_. **(C)** Kinetics of differences between light absorption at 515 nm and absorption at 550 nm, A_515_–A_550_. LS, light scattering; ECS_pmf_, ECS_ΔΨ_, and ECS_ΔpH_ represent electrochromic pigment absorbance shifts proportional to the proton motive force, transmembrane electrical potential, and proton gradient on the thylakoid membrane, respectively.

Local burning is widely used to stimulate the VP in plants (Stanković and Davies, 1996; Hlaváčková et al., 2006; Sukhov et al., 2012, 2014b; Vodeneev et al., 2015); in particular, flames are most commonly used to investigate the influence of electrical signals on photosynthesis (Hlaváčková et al., 2006; Grams et al., 2009; Sukhov et al., 2012, 2014b; Sherstneva et al., 2015, 2016; Surova et al., 2016). Therefore, the VP was induced by burning the tip of the first mature leaf (flame, 3–4 s, ∼1 cm^2^), as shown in **Figure 1A**. This burning was localized and did not change the temperature of the adjacent leaves and stem.

The extracellular measurement of electrical activity was primarily conducted using Ag^+^/AgCl electrodes (RUE “Gomel Measuring Equipment Plant,” Gomel, Belarus), a high-impedance (10^12^ Ω) amplifier IPL-113 (Semico, Novosibirsk, Russia), and a personal computer. First, an electrode was placed on the stem close to the second mature leaf (E_S_), and a second electrode (E_L_) was then placed at the center of the leaflet of this leaf; the distance between E_S_ and E_L_ was 3–4 cm. The electrodes contacted the seedling via ‘Uniagel’ conductive gel (Geltek-Medica, Moscow, Russia). The reference electrode (E_R_) was placed in standard solution (1 mM KCl. 0.5 mM CaCl_2_, 0.1 mM NaCl) surrounding the root.

In a separate experimental series, the influence of a low CO_2_ concentration on the VP parameters was investigated using electrodes consisting of silver wire (0.5-mm diameter) and a pointed tip. The first silver electrode (E_lowCO_2__) was placed at the center of a leaflet in the photosynthesis-measuring head (see below). The second silver electrode (E_control_) was placed at the center of the second leaflet on the same leaf. The reference electrode (E_R_) was placed in standard solution surrounding the root. The CO_2_ concentration was controlled using a photosynthesis measuring system (see below).

### Measurements of Photosynthetic Parameters

A standard system (Heinz Walz GmbH, Effeltrich, Germany) consisting of a portable gas exchange measuring system (GFS-3000), a measuring system for the simultaneous assessment of P700 oxidation and chlorophyll fluorescence (Dual-PAM-100), and a measuring head (Cuvette 3010-Dual) were used to measure photosynthetic parameters.

The photosynthetic parameters were measured under red actinic light (630 nm, 278 μmol m^-2^ s^-1^), a controlled CO_2_ concentration (360 ppm in the most of experiments or approximately 10 ppm (from 7 to 12 ppm) in experiment with low CO_2_ concentration), 67–72% relative humidity, and a temperature of 23°C. The standard functions of the Dual-PAM-100 (light conditions), GFS-3000 (CO_2_ concentration and humidity conditions), and 3010-Dual cuvette (temperature conditions) were used to control the conditions.

The photosynthetic parameters were measured as previously described (Sukhov et al., 2014a, 2015b). The dark (F_0_) and maximal (F_m_) fluorescence yields (Maxwell and Johnson, 2000; Kalaji et al., 2012, 2014) were measured after dark adaptation for 20 min. The maximal change in the P700 signal (P_m_) of PSI, reflecting maximal P700 oxidation (Klughammer and Schreiber, 2008), was measured after preliminary illumination by far red light for 10 s. Later the steady-state (F) and maximal (F′_m_) fluorescence yields in light (Maxwell and Johnson, 2000) and steady-state (P) and maximal (P′_m_) P700 signals in light (Klughammer and Schreiber, 2008) were measured using saturation pulses generated every 10 s. Quantum yield of PSI (ϕ_PSI_) was calculated using the equation ϕ_PSI_ = (P_m_′ - P)/P_m_ (Klughammer and Schreiber, 2008); quantum yield of PSII (ϕ_PSII_) was calculated using the equation ϕ_PSII_ = (F_m_′ - F)/F_m_′ (Maxwell and Johnson, 2000); fluorescence non-photochemical quenching (NPQ) was calculated using the equation NPQ = (F_m_ - F_m_′)/F_m_′ (Maxwell and Johnson, 2000; Kalaji et al., 2012). The CO_2_ assimilation rate (A_CO2_, μmol CO_2_⋅m^-2^⋅s^-1^) was measured using the GFS-3000 system and its software, and the parameter programmatically calculated according to von Caemmerer and Farquhar (1981).

### Analysis of Light Scattering and Electrochromic Shift

A Dual-PAM-100 with P515/535 emitter–detector modules, GFS-3000, and 3010-Dual cuvette (Heinz Walz GmbH, Effeltrich, Germany) were used to measure LS and the ECS.

Light scattering at 530 nm was used to qualitatively estimate the pH in the lumen because it reflected the internal acidification of thylakoids upon light-induced ΔpH formation (Deamer et al., 1967; Murakami and Packer, 1970; Schreiber and Klughammer, 2008). Periodic ‘light-dark’ transitions were used to analyze LS. For each cycle, the duration of illumination by red actinic light was 450 s, and the duration of darkening was 150 s. The magnitude of LS was assessed by measuring the change in absorption with slow relaxation kinetics (∼90–120 s) according to Schreiber and Klughammer (2008). **Figure 1B** shows the methodology used to measure LS. The mean LS magnitude, before VP or a CO_2_ decrease was assumed to be 100%; relative LS were used in the analysis.

Differences between light absorption at 515 and 550 nm were used to analyze the ECS (Schreiber and Klughammer, 2008). Changes in this difference under the light-dark transition with different relaxation kinetics were used to estimate the pmf (ECS_pmf_), ΔΨ (ECS_ΔΨ_), and proton gradient (ECS_ΔpH_). The methods used to estimate the ECS_pmf_, ECS_ΔΨ_, and ECS_ΔpH_ (Avenson et al., 2004; Schreiber and Klughammer, 2008; Bailleul et al., 2010; Klughammer et al., 2013) are shown in **Figure 1C**. The mean electrochromic shift before VP or a CO_2_ decrease was assumed to be 100%; relative ECS_pmf_, ECS_ΔΨ_, and ECS_ΔpH_ values were used for the analysis.

According to several studies (Morita et al., 1982; Sacksteder et al., 2000; Wang et al., 2015) the rapid relaxation of ECS under dark conditions reflects the H^+^-ATP synthase activity because the proton flux through H^+^-ATP synthase is likely the main mechanism underlying proton motive force changes after the onset of darkness. In contrast to most methods of chemical or molecular analysis, measuring rapid ECS relaxation can be used to investigate the dynamics of rapid (seconds and minutes) changes in the H^+^-ATP synthase activity in intact leaves; furthermore, this method is relatively simple. Therefore, we used ECS relaxation measurements to analyze changes in the H^+^-ATP synthase activity after VP or a CO_2_ decrease. The rate constant of rapid ECS relaxation (k_ECS_) was estimated by fitting the first 80 ms of the decay curve with a first-order exponential decay kinetic as the inverse of the decay time constant. Based on the approach of Wang et al. (2015), the k_ECS_ reflected the proton conductivity of H^+^-ATP synthase.

Two variants of periodic ‘light-dark’ transitions were used to analyze the ECS. The ECS_ΔΨ_ and ECS_ΔpH_ were estimated under light:dark conditions of 450 s:150 s; this condition was similar to that used to investigate LS. The k_ECS_ was calculated under light:dark conditions of 50 s:10 s. The ECS_pmf_ was estimated for both light:dark regimens.

The conditions of these measurements were similar to those used for photosynthetic investigations. A photosynthesis measuring system was used to control conditions.

### Statistics

Each measurement was performed on a separate plant. Representative records, mean values, and standard errors were determined and are presented in the figures. Numbers of replicates are shown in the figures. Significant differences were determined according to the Student’s *t*-test.

## Results

### Influence of Burning of Leaf on Photosynthesic Parameters in the Intact Leaf at Atmospheric and Low CO_2_ Concentrations

**FIGURE 2:**
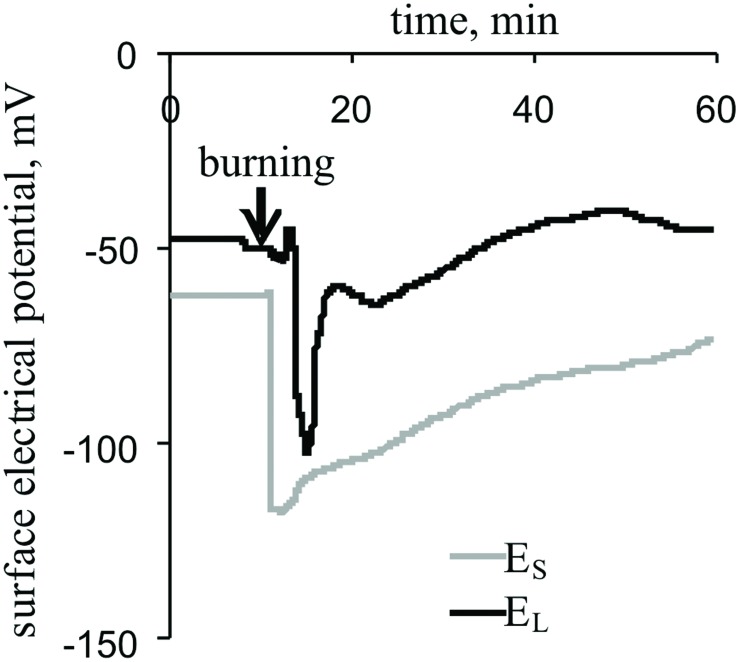
**Changes in the surface electrical potential of the stem near the second leaf (E_S_) and at the center of its leaflet (E_L_), as induced by the burning of the first leaf (*n* = 15)**.

The local burning of the first leaf induced a propagating electrical signal observable in the stems and second leaves of pea plants (**Figure 2**). The mean VP amplitudes were 64 ± 3 mV in the stem and 52 ± 6 mV in the leaf. The average time between the appearance of the VP in the stem and a leaflet at the center of the second leaf was approximately 100 s. The duration of the VP was at least 20–60 min, and the electrical reaction shape was variable.

**FIGURE 3:**
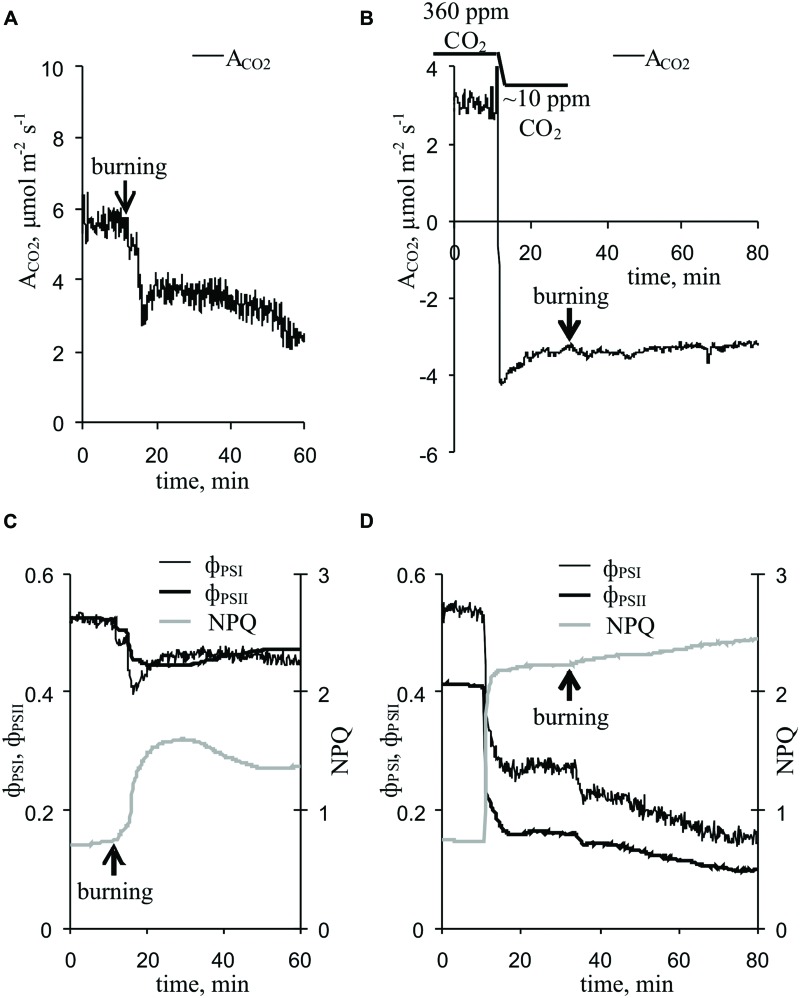
**Changes in the photosynthetic parameters induced by VP at 360 ppm and approximately 10 ppm CO_2_ (*n* = 5–10) (A)** Changes in the A_CO2_ induced by VP at 360 ppm CO_2_. **(B)** Changes in A_CO2_ induced by VP at approximately 10 ppm CO_2_. **(C)** Changes in parameters of light reactions of photosynthesis induced by VP at 360 ppm CO_2_. **(D)** Changes in parameters of light reactions of photosynthesis induced by VP at approximately 10 ppm CO_2_. VP was induced by burning the first mature leaf (arrow).

Upon propagating into the leaf, the VP decreased the CO_2_ assimilation, ϕ_PSI_ and ϕ_PSII_ and increased NPQ (**Figure 3A**). The characteristics of these changes are shown in **Table 1**. Photosynthetic parameters began to change 1-2 min after the start of VP in the leaf. The VP amplitude in the leaf significantly correlated with the magnitudes of changes in the A_CO2_ and NPQ (**Table 1**). Time of beginning of VP in the leaf was significantly correlated with time of beginning of changes in the A_CO2_ and NPQ (**Table 1**). A connection between changes in the A_CO2_ and parameters of light reactions of photosynthesis was also observed (**Table 1**).

**Table 1:** Characteristics of changes in photosynthetic parameters after VP induction and CO_2_ concentration lowering.

	ΔA_CO2_	Δϕ_PSI_	Δϕ_PSII_	ΔNPQ
**Absolute parameter changes**				
VP at 360 ppm CO_2_	-1.9 ± 0.3^∗^, μmol m^-2^ s^-1^	-0.112 ± 0.009^∗^	-0.081 ± 0.01^∗^	0.51 ± 0.10^∗^
CO_2_ concentration lowering	-7.2 ± 0.6^∗^, μmol m^-2^ s^-1^	-0.253 ± 0.017^∗^	-0.301 ± 0.023^∗^	1.63 ± 0.11^∗^
VP at ∼10 ppm CO_2_	-0.3 ± 0.1^∗^^#^, μmol m^-2^ s^-1^	-0.088 ± 0.004^∗^	-0.051 ± 0.006^∗^^#^	0.17 ± 0.08^∗^^#^
**Relative parameter changes, %**				
VP at 360 ppm CO_2_	-31^∗^	-22^∗^	-17^∗^	83^∗^
CO_2_ concentration lowering	-109^∗^	-45^∗^	-59^∗^	203^∗^
VP at ∼10 ppm CO_2_	-4^∗^^#^	-16^∗^	-10^∗^^#^	21^∗^^#^
**Correlation coefficients between the VP amplitude in leaf and the magnitudes of photosynthetic parameter changes**				
VP at 360 ppm CO_2_	-0.67^&^	-0.55	-0.42	0.77^&^
**Correlation coefficients between the initiation time of the VP in leaf and the initiation time of the changes in photosynthetic parameters**				
VP at 360 ppm CO_2_	-0.76^&^	-0.52	-0.57	0.78^&^
**Correlation coefficients between ΔA_CO2_ and the magnitudes of other photosynthetic parameter changes**				
VP at 360 ppm CO_2_	–	0.77^&^	0.65^&^	-0.68^&^

*^∗^p < 0.05 compared with parameter rate at 360 ppm CO_2_, Student t-test*.

*^#^*p* < 0.05 compared with changes in parameter rate induced by VP at 360 ppm CO_*2*_, Student *t*-test*.

*^&^correlation coefficient is significant (*p* < 0.05), Student *t*-test*.

$r\,e\,l\,a\,t\,i\,v\,e\,\;p\,a\,r\,a\,m\,e\,t\,e\,r\,\;c\,h\,a\,n\,g\,e=\frac{a\,b\,s\,o\,l\,u\,t\,e\,\;\;p\,a\,r\,a\,m\,e\,t\,e\,r\,\;c\,h\,a\,n\,g\,e}{p\,a\,r\,a\,m\,e\,t\,e\,r\,\;r\,a\,t\,e\,\;u\,n\,d\,e\,r\,\;c\,o\,n\,t\,r\,o\,l\,\;c\,o\,n\,d\,i\,t\,i\,o\,n}\times 100\%$

*Times of beginning of VP and photosynthetic changes were measured from the moment of burning*.

A decrease in the CO_2_ concentration decreased the CO_2_ assimilation, ϕ_PSI_ and ϕ_PSII_ and increased NPQ (**Figure 3B**, **Table 1**), and these changes were similar to the VP-induced photosynthetic response. The VP-induced photosynthetic response was weak at low CO_2_ concentration (∼10 ppm). All changes, excluding ϕ_PSI_ changes, were significantly lower than those observed at the atmospheric CO_2_ concentration (**Table 1**).

**FIGURE 4:**
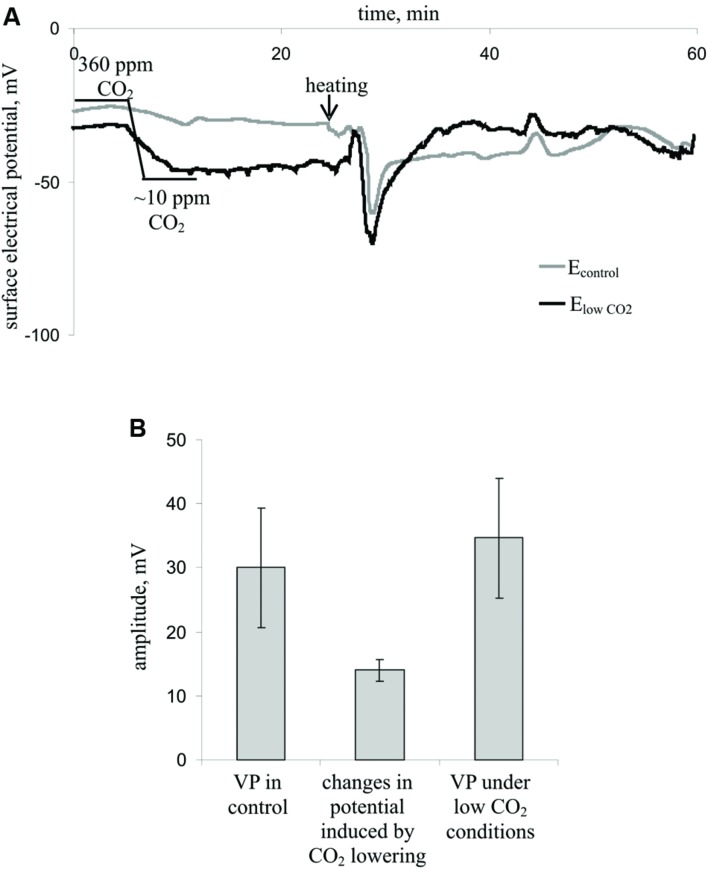
**Influence of decreasing the CO_2_ concentration on the surface membrane potential and VP in leaves (*n* = 7). (A)** Changes in the surface electrical potential in leaflets at approximately 10 ppm CO_2_ concentration (E_lowCO_2__) and in control leaflets (E_control_). **(B)** Mean VP and CO_2_ decrease-induced changes in the surface potential. Significant differences between variants in **Figure 4B** were absent (Student’s *t*-test).

**Figure 4** shows the influence of a decreased CO_2_ concentration on the surface membrane potential and VP parameters. Decreasing the CO_2_ concentration decreased the surface potential (**Figure 4A**) by approximately 15 mV (**Figure 4B**) but did not influence the VP amplitude (**Figures 4A,B**). Moreover, the VP amplitudes under low CO_2_ conditions and control conditions strongly correlated (correlation coefficient was 0.77, *p* < 0.05), whereas the change in the surface potential after decreasing the CO_2_ concentration and VP amplitude did not correlate (data not shown). Notably, the VP measured by silver electrodes (**Figure 4A**) did not significantly differ from the VP measured by Ag^+^/AgCl electrodes in leaves (**Figure 2**). Differences in amplitudes were also insignificant.

### Influence of Burning of Leaf on Magnitude and Relaxation of Electrochromic Pigment Absorbance Shift

**FIGURE 5:**
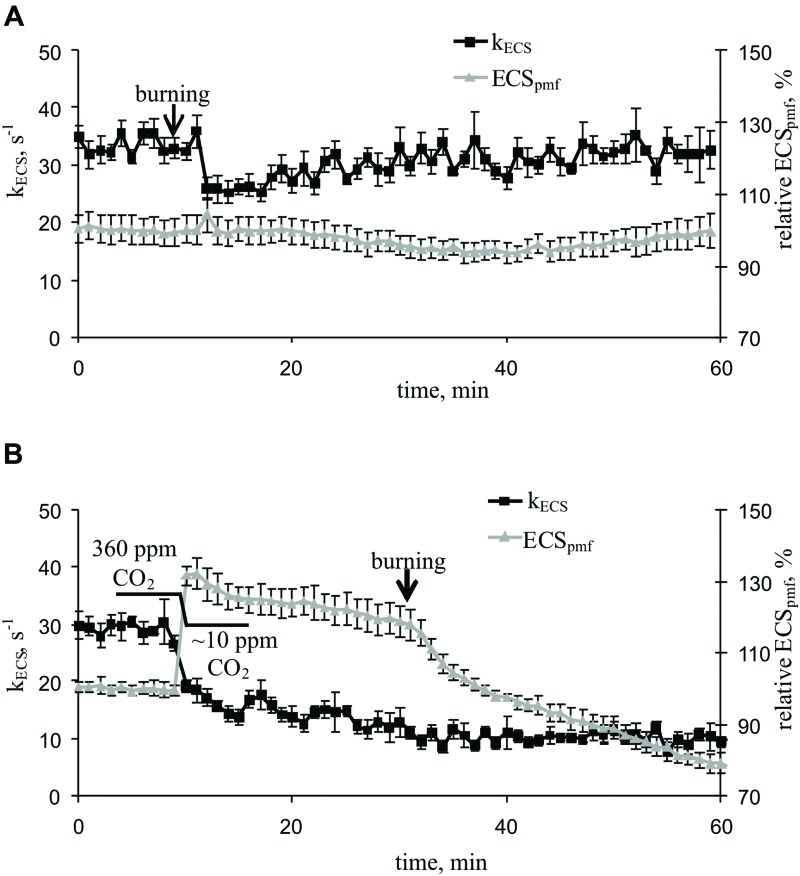
**Changes in the rate constant of the rapid relaxation of the electrochromic pigment absorbance shift (k_ECS_), reflecting the proton conductivity of H^+^-ATP synthase, and the relative electrochromic shift, reflecting the proton motive force (relative ECS_pmf_) induced by VP at 360 ppm **(A)** and approximately 10 ppm **(B)** CO_2_ (*n* = 5–6).** The mean ECS_pmf_ magnitudes prior to VP or a decrease in the CO_2_ level were assumed to be 100%. Points were measured every 60 s (50 s light, 10 s dark). The VP was induced by burning the first mature leaf (arrow).

Local burning and, probably, propagation of burning-induced VP decreased the rate constant of rapid ECS relaxation (k_ECS_, **Figure 5A**, **Table 2**), reflecting the proton conductivity of the H^+^-ATP synthase decrease (Wang et al., 2015). The minimum of rate constant (∼70% of the initial rate) was observed 2–7 min after the induction of the VP. Decreasing the CO_2_ concentration (**Figure 5B**, **Table 2**) also decreased the k_ECS_, and the minimal value was approximately 40% of the initial rate. However, the VP did not decrease the k_ECS_ under low CO_2_ concentration.

**Table 2:** Relative changes in the ECS and LS parameters after VP induction and CO_2_ concentration decrease.

	VP at 360 ppm CO_2_	CO_2_ concentration lowering	VP at ∼10 ppm CO_2_
Δk_ECS_, %	-29^∗^	-59^∗^	-6^#^
ΔECS_pmf_, %	-12^∗^	+41 ^∗^	-46^∗^^#^
ΔECS_ΔΨ_, %	-11	+18	-48^∗^^#^
ΔECS_ΔpH_, %	-18^∗^	+108 ^∗^	-45^∗^^#^
ΔLS, %	+148^∗^	+139 ^∗^	+90^∗^

*^∗^*p* < 0.05 compared with parameter rate at 360 ppm CO_*2*_, Student *t*-test*.

*^#^*p* < 0.05 compared with changes in parameter rate induced by VP at 360 ppm CO_*2*_, Student *t*-test.*

$r\,e\,l\,a\,t\,i\,v\,e\,\;p\,a\,r\,a\,m\,e\,t\,e\,r\,\;c\,h\,a\,n\,g\,e=\frac{a\,b\,s\,o\,l\,u\,t\,e\,\;\;p\,a\,r\,a\,m\,e\,t\,e\,r\,\;c\,h\,a\,n\,g\,e}{p\,a\,r\,a\,m\,e\,t\,e\,r\,\;r\,a\,t\,e\,\;u\,n\,d\,e\,r\,\;c\,o\,n\,t\,r\,o\,l\,\;c\,o\,n\,d\,i\,t\,i\,o\,n}\times 100\%$

The VP induced a weak decrease in the relative ECS_pmf_, reflecting the VP-induced changes in the proton motive force. **Figure 5B** shows that decreasing the CO_2_ level increased the relative ECS_pmf_, whereas the VP decreased the proton motive force at low CO_2_ concentration. The magnitude of the VP-induced ECS_pmf_ decrease at a low CO_2_ concentration was greater than that observed at atmospheric CO_2_ concentration.

### Influence of Burning of Leaf on ΔpH- and ΔΨ-Dependent Components of Electrochromic Pigment Absorbance Shift and Light Scattering

**FIGURE 6:**
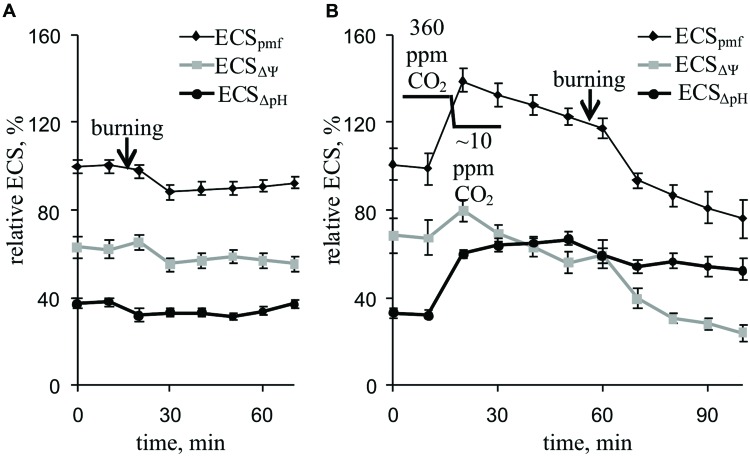
**Changes in the relative ECS_pmf_, ECS_ΔΨ_, and ECS_ΔpH_ induced by VP at 360 ppm **(A)** and approximately 10 ppm **(B)** CO_2_ (*n* = 6–8).** The mean ECS_pmf_ magnitudes prior to VP or a decrease in the CO_2_ were assumed to be 100%. Points were measured every 600 s (450 s light, 150 s dark). The VP was induced by burning the first mature leaf (arrow).

Local burning and, probably, propagation of burning-induced VP changed the relative ECS_pmf_, ECS_ΔΨ_, and ECS_ΔpH_ reflecting proton motive force, ΔΨ, and ΔpH across the thylakoid membrane (**Figure 6A**; **Table 2**). Under a light:dark regimen of 450-s light:150-s dark, the VP decreased the ECS_pmf_, similar to the proton motive force decreases observed under a 50-s light:10-s dark regimen. The ECS_ΔΨ_ and ECS_ΔpH_ also decreased after VP induction, which reflected a reduction in the ΔΨ and ΔpH. Decrease of the CO_2_ concentration (**Figure 6B**, **Table 2**) increased the ECS_pmf_ and ECS_ΔpH_ but only weakly influenced the ECS_ΔΨ_. The VP significantly decreased all investigated parameters at low CO_2_ concentration. The magnitudes of the VP-induced ECS_pmf_, ECS_ΔΨ_, and ECS_ΔpH_ decreases at low CO_2_ concentration were larger than those under control conditions.

**FIGURE 7:**
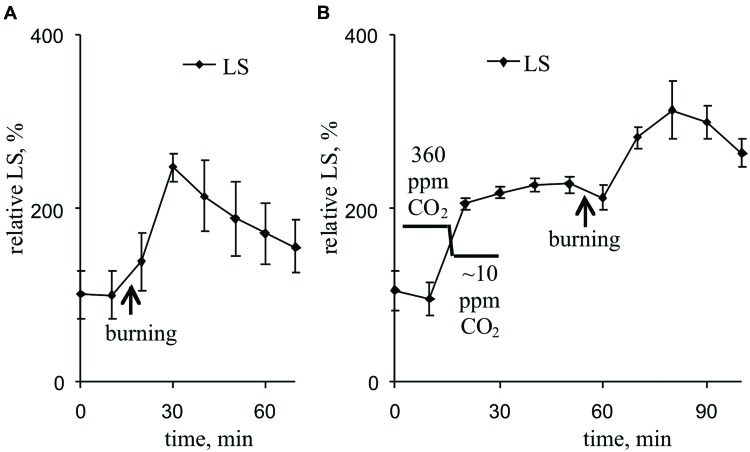
**Changes in the relative LS induced by VP at 360 ppm **(A)** and approximately 10 ppm **(B)** CO_2_ (*n* = 6–8).** The mean LS magnitudes prior to VP or a decrease in the CO_2_ were assumed to be 100%. Points were measured every 600 s (450 s light, 150 s dark). The VP was induced by burning the first mature leaf (arrow).

**Figure 7A** and **Table 2** show that the VP transiently increased LS, which likely reflects a pH decrease in the thylakoid. Maximum LS growth occurred approximately 14 min after VP induction. Decreasing the CO_2_ concentration also increased LS (**Figure 7B**, **Table 2**). The VP also increased LS at low CO_2_ concentration, but this LS growth was less pronounced than that observed under control conditions.

## Discussion

Local burning induced VP propagation (**Figures 2** and **4**) and elicited a photosynthetic response in undamaged pea leaves under red actinic light (**Figure 3A**, **Table 1**). VP propagation is based on hydraulic and/or chemical signal propagation (Malone, 1994; Stahlberg and Cosgrove, 1996; Mancuso, 1999; Vodeneev et al., 2012, 2015; Sukhov et al., 2013); therefore, the first question is ‘does the VP induce a photosynthetic response or can hydraulic and/or chemical signals influence photosynthesis without a VP?’ Literature data (Grams et al., 2007) show that electrical and hydraulic signals can have distinct effects on leaf gas exchange. Our results showed that the VP amplitude was strongly correlated with the magnitudes of local burning-induced changes in CO_2_ assimilation and NPQ, and the initiation time of the VP in the leaf was strongly correlated with the initiation time of changes in these photosynthetic parameters (**Table 1**). These correlations can be explained by (i) hydraulic and/or chemical signals having very similar effects on electrical activity and photosynthesis, (ii) the effect of the photosynthetic response on electrical activity and (iii) the effect of the VP on photosynthesis. There are a number of arguments supporting the last supposition. First, the VP was propagated into undamaged leaves 1–2 min before the initiation of the photosynthetic response. Second, our previous results (Sukhov et al., 2014a; Sukhov, 2016) showed that imitation of a VP-connected proton influx (treatment of protonophores) caused a photosynthetic response in pea leaves, and the response was similar to the response induced by a VP. Third, the VP-induced photosynthetic response was very similar to the AP-induced response (Krupenina and Bulychev, 2007; Pavlovič et al., 2011), but AP is not connected with hydraulic or chemical signals (Fromm and Lautner, 2007). Thus, we suppose that VP induces a photosynthetic response in peas under red light.

According to previous studies (Krupenina and Bulychev, 2007; Pavlovič et al., 2011; Sukhov et al., 2012, 2014a,b, 2015b; Sukhov, 2016), electrical signal-induced inactivation of the dark reactions of photosynthesis is the main initial mechanism of the photosynthetic response. The following hypothetical chain of events was previously proposed to explain the photosynthetic response (Pavlovič et al., 2011; Sukhov et al., 2012, 2014a): electrical signals (AP or VP) → inactivation of the dark reactions of photosynthesis → an increase in the ATP:ADP ratio → inactivation of H^+^-ATP synthase → a decrease in H^+^ flux from the lumen to the stroma → alkalization of the chloroplast stroma and acidification of its lumen → an increase in the proton electrochemical gradient across thylakoid membranes → inactivation of the light reactions of photosynthesis.

The results of the current study show that inactivation of the dark reactions is involved in the initiation of the photosynthetic response. There are three groups of arguments to support this, as follows. (i) The magnitude of VP-induced A_CO2_ inactivation was significantly correlated with the magnitudes of changes in the parameters of the light reactions of photosynthesis. (ii) Artificial suppression of the dark reactions of photosynthesis, caused by lowering the CO_2_ concentration, decreased the quantum yields of PSI and PSII and increased NPQ (**Figure 3D**, **Table 1**); these changes were similar to VP-induced changes (**Figure 3C**, **Table 1**). (iii) The magnitudes of VP-induced changes in the parameters of photosynthetic light reactions were decreased at a low CO_2_ concentration (**Figure 3D**, **Table 1**). This effect was not connected with changes in VP parameters under a low CO_2_ concentration because the amplitudes and shapes of the VPs were similar at low and atmospheric CO_2_ concentrations (**Figure 4**). Thus, the photosynthetic response in peas under red light is primarily initiated by inactivation of the dark reactions of photosynthesis; i.e., the mechanisms of the VP-induced photosynthetic responses under blue light (Sukhov et al., 2014a,b, 2015b) and red light (current study) are similar.

However, the strong suppression of dark reaction inactivation under low CO_2_ concentrations did not strongly inactivate the responses of the photosynthetic light reactions, especially those of ϕ_PSI_ and ϕ_PSII_ (the magnitudes of the changes were 79% and 66% from their magnitudes under the atmospheric CO_2_ concentration). This result shows that the influence of VP on the light reactions of photosynthesis can be observed without inactivating the dark reactions under red light; i.e., additional pathways are involved. It is also in good agreement with our previous results in peas under blue light (Sukhov et al., 2014a, 2015b). According to our previous hypothesis (Sukhov et al., 2012, 2014a; Sukhov, 2016), proton flux from the chloroplast to the stroma and lumen, and their acidification, may be an additional mechanism by which VP affects the light reactions; however, this supposition needs experimental analysis.

We also investigated the effect of VP on the activity of H^+^-ATP synthase using ECS relaxation (Morita et al., 1982; Sacksteder et al., 2000; Schreiber and Klughammer, 2008; Wang et al., 2015). Our results indicated that the VP decreased the rate constant of ECS relaxation (**Figure 5A**, **Table 2**), reflecting a decrease in the proton conductivity of H^+^-ATP synthase (Wang et al., 2015). Artificial suppression of the dark reactions of photosynthesis, by lowering the CO_2_ concentration, also decreased the rate constant of ECS relaxation, and the VP did not induce significant changes in k_ECS_ under a low CO_2_ concentration (**Figure 5B**, **Table 2**). Our results experimentally support the hypothesis that H^+^-ATP synthase activity is decreased after electrical signal-induced suppression of the dark reactions of photosynthesis (Pavlovič et al., 2011; Sukhov et al., 2012, 2014a).

The decrease in H^+^-ATP synthase activity after suppression of the dark reactions of photosynthesis probably results in an increase of the proton motive force (proton electrochemical gradient) across thylakoid membranes (Pavlovič et al., 2011; Sukhov et al., 2012, 2014a, 2015b). Here, artificial suppression of the dark reactions of photosynthesis increased the ECS_pmf_, ECS_ΔpH_, and ECS_ΔΨ_ (**Figure 6B**, **Table 2**), which reflect the proton motive force, proton gradient, and ΔΨ across thylakoid membranes, respectively (Avenson et al., 2004; Schreiber and Klughammer, 2008; Bailleul et al., 2010). LS and NPQ, which are connected with lumen acidification (Deamer et al., 1967; Murakami and Packer, 1970; Ruban et al., 1993; Maxwell and Johnson, 2000; Müller et al., 2001; Schreiber and Klughammer, 2008; Goss and Lepetit, 2015), were also stimulated after lowering the CO_2_ concentration (**Figures 3D** and **7B**, **Tables 1** and **2**).

However, our results showed (**Figures 5A** and **6A**, **Table 2**) that VP moderately decreased the ECS_pmf_, ECS_ΔpH_, and ECS_ΔΨ_ under an atmospheric CO_2_ concentration; i.e., VP probably reduce the proton motive force, proton gradient, and ΔΨ across thylakoid membranes.

Two *a priori* hypotheses can be proposed for the VP-induced ΔpH decrease: (i) an increase in the internal pH of the thylakoid lumen or (ii) a decrease in the pH of the chloroplast stroma. The first hypothesis is not supported by the experimental data. Firstly, we found that VP induction increased LS (**Figure 7A**, **Table 2**), reflecting the internal acidification of the thylakoids (Deamer et al., 1967; Murakami and Packer, 1970; Schreiber and Klughammer, 2008), i.e., the luminal pH is probably decreased after VP propagation. Secondly, the VP-induced increase in NPQ (**Figure 3C**, **Table 1**) supports a decrease of luminal pH because lumen acidification is known to increase NPQ (Ruban et al., 1993; Maxwell and Johnson, 2000; Müller et al., 2001; García-Plazaola et al., 2012; Goss and Lepetit, 2015). Thirdly, a decrease of H^+^-ATP synthase activity (a decrease of proton eﬄux from the lumen to the stroma) can also stimulate lumen acidification.

The second hypothesis implies that the ΔpH decrease is connected with a decrease in the pH of the chloroplast stroma. This hypothesis explains the simultaneous ΔpH decrease (reduction in ECS_ΔpH_) and luminal pH decrease (LS and NPQ increases), i.e., it is very probable. Additionally, the second hypothesis is well supported by literature data, which demonstrate that VP generation is accompanied by H^+^-ATPase inactivation in the plasma membrane (Sukhov et al., 2013; Katicheva et al., 2014; Vodeneev et al., 2015) and a decrease in the pH of the cytoplasm (Grams et al., 2009; Sukhov et al., 2014a; Sherstneva et al., 2015, 2016); notably, this decrease was observed in pea seedlings (Sukhov et al., 2014a; Sherstneva et al., 2016). The acidification of the cytoplasm can contribute to proton flux into the stroma through different H^+^-transporting systems in the membrane envelope (Peters and Berkowitz, 1991; Wu and Berkowitz, 1992; Song et al., 2004). Moreover, a stromal pH decrease can contribute to a decrease of the luminal pH via proton transport through the photosynthetic electron-transport chain (Allen, 2003), i.e., this decrease may participate in VP-induced lumen acidification.

The VP-induced decrease in the proton motive force is likely connected to the ΔpH decrease (**Figure 6A**, **Table 2**). However, the tendency of ΔΨ to decrease after a VP, which also decreases the proton motive force, may be related to the acidification of the thylakoid lumen because a luminal pH decrease can suppress the photosynthetic electron-transport chain activity (Kramer et al., 1999; Tikhonov, 2013, 2014).

Variation potential-induced stroma and lumen acidification can inactivate the light reactions of photosynthesis. Decrease in the stromal pH is known to change ferredoxin-NADP^+^ reductase localization (Benz et al., 2010), which suppresses electron flow through PSI. Moreover, decrease in the lumen pH is well known to stimulate NPQ (Ruban et al., 1993; Maxwell and Johnson, 2000; Müller et al., 2001) and directly suppresses photosynthetic electron-transport chain activity (Kramer et al., 1999; Tikhonov, 2013, 2014). Both processes decrease the quantum yields of the photosystems, i.e., inactivate photosynthesis. It is possible that these mechanisms participate in additional pathways by which electrical signals affect the light reactions of photosynthesis because an electrical signal-induced photosynthetic response can develop without inactivation of the dark reactions of photosynthesis (Sukhov et al., 2012, 2014a, 2015b; Vredenberg and Pavlovič, 2013; Sukhov, 2016).

## Author Contributions

VS conceived and supervised the project. VS and VV designed the experiments. LS, EM, and OS performed the experiments. VS, LS, and VV analyzed the data. VS and VV wrote the manuscript. All authors participated in the discussions of the results and the preparation of the manuscript.

## Conflict of Interest Statement

The authors declare that the research was conducted in the absence of any commercial or financial relationships that could be construed as a potential conflict of interest.
